# Diabetes and beta-adrenergic blockage are risk factors for metastatic prostate cancer

**DOI:** 10.1186/s12957-017-1117-4

**Published:** 2017-02-21

**Authors:** Malte Krönig, Christian Haverkamp, Antonia Schulte, Laura Heinicke, Kathrin Schaal, Vanessa Drendel, Martin Werner, Ulrich Wetterauer, Wolfgang Schultze-Seemann, Cordula Annette Jilg

**Affiliations:** 1 Department of Urology, Uniklinikum Freiburg, Hugstetter Strasse 55, 79106 Freiburg, Germany; 2 Institut of Clinical Pathology, Uniklinikum Freiburg, Breisacher Strasse 115a, 79106 Freiburg, Germany; 3 IT-Department, Uniklinikum Freiburg, Agnesenstrasse 6-8, 79106 Freiburg, Germany; 4 Department of Urology, University of Freiburg Medical Centre, Hugstetter Strasse 55, 79106 Freiburg, Germany

**Keywords:** Prostate cancer, Metastasis, Diabetes, Beta-adrenergic blockage

## Abstract

**Background:**

We evaluated the influence of comorbidity inferred risks for lymph node metastasis (pN1) and positive surgical margins (R1) after radical prostatectomy in order to optimize pretherapeutic risk classification.

We analyzed 454 patients after radical prostatectomy (RP) between 2009 and 2014. Comorbidities were defined by patients’ medication from our electronic patient chart and stratified according to the ATC WHO code. Endpoints were lymph node metastasis (pN1) and positive surgical margins (R1).

**Results:**

Rates for pN1 and R1 were 21.4% (97/454) and 29.3% (133/454), respectively. In addition to CAPRA and Gleason score, we identified diabetes as a significant medication inferred risk factor for pN1 (OR 2.9, *p* = 0.004/OR 3.2, *p* = 0.001/OR 3.5, *p* = 0.001) and beta-blockers for R1 (OR 1.9, *p* = 0.020/OR 2.9, *p* = 0.004). Patients with diabetes showed no statistically significant difference in Gleason score, CAPRA Score, PSA, and age compared to non-diabetic patients.

**Conclusions:**

We identified diabetes and beta1 adrenergic blockage as significant risk factors for lymph node metastasis and positive surgical margins in prostate cancer (PCa). Patients at risk will need intensive pretherapeutic staging for optimal therapeutic stratification.

**Electronic supplementary material:**

The online version of this article (doi:10.1186/s12957-017-1117-4) contains supplementary material, which is available to authorized users.

## Background

Prostate cancer (PCa) is the second most common cancer and third most leading cause of death of men in the western world [1]. Correct risk stratification is crucial for optimal of high-risk patients and avoiding overtreatment in low-risk patients. Risk stratification is based on histologic analysis of invasive prostate biopsies, which are indicated by elevated prostate-specific antigen (PSA) levels or suspicious digital rectal examination (DRE) findings. Several risk classification tools exist for pretherapeutic stratification such as Kattan normograms [2], D’Amico score [3, 4] or CAPRA (Cancer of Prostate Risk Assessment) score [5–7]. All scores are based primarily on PSA levels (ng/ml), Gleason score, and age (years) and have biochemical recurrence within 5 years after radical prostatectomy as primary endpoint. The mentioned scores stratify patients into low-, intermediate-, and high-risk groups. However, identifying patients with preexisting lymph node metastases (cN1) or non-organ defined tumors (R1) remains difficult, but these parameters significantly determine the therapeutic strategy. Imaging which provide detailed information for cN status and R status, such as magnetic resonance imaging (MRI) or positron emission computer tomography (PET/CT) (e.g., PSMA-PET/CT), cannot be performed in every patient for economic and availability reasons. cN1 status would require extended lymphadenectomy during radical prostatectomy or extended field radiation in primary radiation therapy. Extended staging using MRI is recommended for high-risk patients with PSA > 10 ng/ml or Gleason score ≥ 8–10 [8] only in the European guidelines for prostate cancer. In order to find further risk stratifier, comorbidities have come into the focus with possible implications on cancer genesis, stage, progression, and therapy [9, 10]. Data are still conflicting, and exact mechanism regarding stage and prognosis are not understood. Among the comorbidities, diabetes is one of the better examined and most common diseases with an estimated 269 million people affected worldwide [11]. Interestingly, a reduced risk for developing PCa has been described for diabetes patients [11]; however, little is known about the impact on cancer stage. We have therefore used our electronic patient chart to generate the prostate cancer patients’ comorbidity profile by the self-medication during hospitalization for radical prostatectomy. We assessed the comorbidity profile’s impact on cancer stage at diagnosis represented by R status and pN status as the key determinants of primary and adjuvant therapeutical strategy.

## Methods

We included 454 patients with prostate cancer, who were treated with radical prostatectomy (RP) between 2009 and 2014 (median age 66 years, interquartile range (IQR 61.0–71.0). The same surgical team operated these patients with equal experience. Comorbidities were defined by patients’ self-medication during hospitalization for radical prostatectomy. Medications were generated from our electronic patient chart (Meona©) and stratified according to the ATC (Anatomic Therapeutic Chemical) WHO code at the time of the RP.

### ATC code

Anatomical Therapeutic Chemical (ATC) classification system divides the active substances into different groups according to the organ or system on which they act and their therapeutic, pharmacological, and chemical properties. Drugs are classified in groups at five different levels. The drugs are divided into 14 main groups (1^st^ level), with pharmacological/therapeutic subgroups (2^nd^ level). The 3^rd^ and 4^th^ levels are chemical/pharmacological/therapeutic subgroups, and the 5^th^ level is the chemical substance [12].

### CAPRA score

Cancer of the Prostate Risk Assessment CAPRA [13–16] score was used for pretherapeutic risk classification. The score includes PSA at diagnosis (ng/ml), Gleason pattern of the biopsy (primary/secondary), age (years), and positive biopsy cores (percent of total number of biopsies) as variables (Additional file 1: Table S1), which are weighted differently according to the value.

### Endpoints

Endpoints were lymph node metastasis (pN1) and positive surgical margins (R1) after radical prostatectomy based on the histopathologic analysis of the radical prostatectomy specimens including lymph nodes. Specimens were routinely processed, and analysis was performed on paraffin embedded, cut, and H&E-stained samples.

### Statistics

Descriptive statistics was done by calculating mean ± standard deviation (SD), median, and interquartile range (IQR). Logistic and linear regression analyses were used for identifying risk factors using SPSS© software (SPSS statistics 22, IBM) calculated as odds ratio (OR) and *p* value.

## Results

Four hundred fifty-four prostate cancer patients after radical prostatectomy were analyzed in this study. Patients’ median age was 66.0 years (IQR 61.0–70.0) and median iPSA 8.55 ng/ml (IQR 5.67–14.43). 18.5% (85/454), 39.88% (181/454), and 41.41% (188/454) showed CAPRA score 1–2, 3–5 and 6–10, respectively. Histopathology from prostatectomy and lymphadenectomy showed in 11.7% (53/454), 62.33% (283/454), and 25.99% (118/454) Gleason score 6, 7, and 8–10 (Table 1). Rates for pN1 and R1 were 21.37% (97/454) and 29.30% (133/454), respectively (Table 1).

**Table 1:** Patients’ characteristics (*n* = 454)

Age at radical prostatectomy (years)	
Mean/±SD/median/IQR	65.3/6.7/66.0/61.0–70.0
PSA at radical prostatcetomy (ng/ml)	
Mean/±SD/median/IQR	13.8/24.4/8.6/5.6–14.4
Gleason score at biopsy (*n*)	
% (*n*/total)	
8–10	25.9 (118/454)
7	62.3 (283/454)
6	11.7 (53/454)
Gleason score at radical prostatectomy (*n*)	
% (*n*/total)	
8–10	21.2 (96/454)
7	60.6 (275/454)
6	18.3 (83/454)
T stage at biopsy (*n*)	
% (*n*/total)	
4	1.9 (9/454)
3	36.8 (167/454)
2	61.2 (278/454)
T stage at radical prostatectomy (*n*)	
% (*n*/total)	
4	0.7 (3/454)
3	34.8 (158/454)
2	64.3 (292/454)
CAPRA score at biopsy (*n*)	
% (*n*/total)	
6–10 (high risk)	41.4 (188/454)
3–5 (intermediate risk)	39.8 (181/454)
1–2 (low risk)	18.5 (85/454)
Positive lymph nodes (N+) at radical prostatectomy (*n*)	
% (*n*/total)	21.3 (97/545)
Positive surgical margin (R+) at radical prostatectomy (*n*)	
% (*n*/total)	29.3 (133/454)

*PSA* prostate specific antigen, *CAPRA* Cancer of the Prostate Risk Assessment

A median of 2 (IQR 1–4) medications from 2 (IQR 1–4) comorbidity level 1 classes were taken per patient. 14.9% (68/454), 54.2% (246/454), 29.7% (135/454), and 1.1% (5/454) of the patients took 0, 1–3, 4–9, and 10–15 medications (Table 5). From clinical parameters such as age (years), PSA (ng/ml), Gleason score, and CAPRA score, we identified CAPRA score and Gleason score as significant risk factors for N1 (OR 3.200, *p* = 0.001/OR 3.454, *p* = 0.001) and CAPRA score for R1 (OR 2.916, *p* = 0.004) (Table 4). Patients with diabetes showed no statistically significant difference in Gleason score (*p* = 0.499), CAPRA score (*p* = 0.495), PSA (*p* = 0.668), and age (*p* = 0.537) compared to non-diabetic patients.

Patients took 157 different types of medications from 9 major comorbidity classes according to the ATC code level 1 [12] (Table 2). 14.89% (68/454), 54.19% (246/454), 29.74% (135/454), and 1.1% (5/454) took 0, 1–3, 4–9, and greater 10 medications at time of radical prostatectomy. Patients took medication for cardiovascular system (C) 68.28% (310/454), alimentary tract (A) 33.70% (153/454), blood system (B) 27.53% (125/454), genitourinary system (G) 12.78% (58/454), hormonal system (H) 10.35% (47/454), nervous system (N) 8.81% (40/454), respiratory system (R) 3.52% (16/454), sensory system (S) 3.08% (14/454), and immune system (L) 0.66% (3/454) (Table 2).

**Table 2:** Characteristics of comorbidities (ATC code level 1, distribution and regression analyses)

ATC code level 1	Distribution	Positive surgical margin (R1)	Lymph node metasasis (N1)
	% (*n*/total)	OR/*p* value	OR/*p* value
Cardiovascular system C	68.28 (310/454)	1.164/0.531	0.904/0.715
Alimentary tract A	33.70 (153/454)	0.864/0.525	1.370/0.203
Blood system B	27.53 (125/454)	1.068/0.789	1.927/0.63
Urinary system G	12.78 (58/454)	0.748/0.382	1.263/0.480
Hormonal system H	10.35 (47/454)	1.356/0.355	1.203/0.621
Nervous system N	8.81 (40/454)	1.662/0.148	1.214/0.617
Respiratory system R	3.52 (16/454)	1.305/0.622	0.431/0.283
Sensory system S	3.08 (14/454)	2.284/0.139	0.913/0.894
Immune system L	0.66 (3/454)	1.216/0.875	0.000/0.999

ATC level 1: level 1 of the Anatomic Therapeutic Chemical (ATC) code describes anatomic organ systems; *OR* odds ratio

Analysis of the ATC code on level 2 showed top 10 medications to be renin angiotensin system (C09) 44.49% (202/454), beta-blockers (C07) 32.28% (147(454), antithrombosis (B01) 27.53% (125/454), lipid modifyers (C10) 26.21% (110/454), acid disorders (A02) 23.57% (107/454), calcium channel blockers (C08) 17.18% (78/454), diuretics (C03) 14.32% (65/454), urologicals (G04) 12.78% (58/454), thyroid therapy (H03) 10.13%(46/454), and diabetes (A10) 9.25% (42/454) (Table 3).

**Table 3:** Characteristics of comorbidities (ATC code level 2, distribution and regression analyses)

ATC code level 2	Comorbidity system	Distribution	Positive surgical margin (R1)	Lymph node metastasis (N1)
		% (*n*/*n*)	OR/*p* value	OR/*p* value
C09	Renin angiotensin system	44.5 (202/454)	0.999/0.997	1.344/0.255
C07	Beta-blockers	32.3 (147(454)	*1.929/0.020*	0.953/0.878
B01	Antithrombosis	27.5 (125/454)	1.063/0.829	1.821/0.52
C10	Lipid modifiers	26.2 (110/454)	0.867/0.616	0.982/0.995
A02	Acid disorders	23.6 (107/454)	0.954/0.860	1.127/0.679
C08	Calcium channel blockers	17.2 (78/454)	0.702/0.311	0.523/0.126
C03	Diuretics	14.3 (65/454)	1.174/0.626	0.779/0.510
G04	Urologicals	12.8 (58/454)	0.653/0.232	1.346/0.387
H03	Thyroid therapy	10.1 (46/454)	1.227/0.557	1.253/0.559
A10	Diabetes	9.3 (42/454)	1.092/0.811	*2.869/0.004*
A07	Diarrhea, inflammation	5.1 (23/454)	0.240/0.074	0.515/0.387
N06	Psychoanaleptics	4.4 (20/454)	1.172/0.761	0.761/0.653
C01	Cardiac therapy	3.9 (18/454)	0.559/0.346	0.550/0.396
R03	Anti asthmatics	3.3 (15/454)	1.009/0.989	0.143/0.148
N05	Psycholeptics	3.1 (14/454)	1.114/0.860	2.254/0.177
C02	Antihypertensives	2.6 (12/454)	0.220/0.158	1.459/0.619
N03	Antiepileptics	1.8 (8/454)	6.048/0.051	2.824/0.306
S01	Opthalmologicals	1.5 (7/154)	2.560/0.334	0.755/0.875
A03	Functional gastroint, disorders	0.7 (3/454)	2.778/0.476	1.078/0.963
L04	Immunosuppression	0.7 (3/454)	3.537/0.361	0.0/0.999
N04	Anti parkinson drugs	0.7 (3/454)	3.956/0.276	0.0 / 0.999
A01	Stomatologic disorders	0.4 (2/454)	0.0/0.999	1.770/0.770
R01	Nasal corticoides	0.4 /2/454)	0.0/0.999	10.577/0.287
Subgroupanalysis				
Diabetes	Insulin	4.2 (19/454)	–	1090/0.882
	Metformin	7.7 (35/454)	–	*2.989/0.009*
Beta-blockers	Carvedilol	1.9 (9/454)	0.341/0.314	–
	Metoprolol	8.9 (40/454)	*2.400/0.010*	–
	Bisoprolol	14.8 (67/454)	1.202/0.533	–
	Nebivolol	2.4 (11/454)	2.884/0.090	–

ATC level 2: level 2 of the Anatomic Therapeutic Chemical (ATC) code describes anatomic or chemical systems within the body or in specific organs; *OR* odds ratio; italic = statistically significant OR

Logistic regression analysis on ATC code level 1 did not show significantly increased risk for pN1 or R1 status whereas regression analysis on ATC code level 2 did show significantly increased risk for pN1 in patients with diabetes (OR 2.869, *p* = 0.004) and beta1 blockers for R1 (OR 1.929, *p* = 0.020). Subgroup analysis showed significantly increased risk for N1 in patients taking metformin (OR 2.989, *p* = 0.010) and R1 for patients taking beta1 selective blocker metoprolol (OR 2.400, *p* = 0.010). The number of medications per patient and the number of medicated organ systems per patient did not show statistically significant risk increase for either N1 or R1 (Table 3).

## Discussion

In this study, we identified diabetes (OR 2.869) and beta-blockage (OR 1.929) as significant risk factors for the existence of lymph metastases (pN1) and non-organ confined (R1) prostate cancer in patients at radical prostatectomy. The comorbidity profile of each patient was defined as the self-medication, which is not associated to hospitalization. More comorbidities might have been present within the cohort, which were missed by this method. However, our methods allow for a detailed analysis of the comorbidities on the organ system level down to single medications utilizing the WHO ATC code. Within the diabetes group, e.g., we identified metformin and in the beta1 selective blockage group metoprolol as single risk factors for pN1 and R1, respectively.

Comorbidities have been associated with tumor genesis and progression, results however remain controversial. Several studies show that the effect can be protective or associated with increased cancer risk in different organs [17]. Especially, diabetes and beta-adrenergic signaling have been associated with carcinogenesis for a long time.

Lipscomb et al. [18] showed that diabetic women (*n* = 6115) have an increased risk (OR 1.16) for developing lymph node positive breast cancer. Obesity or increased BMI are not associated with increased risk for lymph node metastases [19] in breast cancer. We could not identify a study, which specifically analyzed the association between diabetes and nodal status of prostate cancer in a PubMed search until today.

Several studies describe a medication inferred decreased risk for developing prostate cancer: Margel et al. [20] showed in 12,000 prostate cancer patients that, e.g., metformin users had decreased risk (OR 0.66) for developing prostate cancer. Preston et al. [21] showed in 120,000 diabetic men that metformin was not associated with decreased risk for developing prostate cancer in general or high-grade prostate cancer. A meta-analysis by Bansal et al. [22] concluded from 8.1 million patients including 120,000 prostate cancer patients that diabetes significantly lowers the risk (RR 0.86) for developing prostate cancer. Li et al. [23] report opposite results in a cohort of 22,000 men with increased risk for developing high-grade prostate cancer in men with diabetes.

Despite the high numbers of studies showing a protective effect of diabetes on cancer genesis, little is known about the influence of diabetes in men with prostate cancer. Also, on the mechanistic level, it was shown that diabetes alters the lymphatic vessel architecture and promotes vessel evasion of the tumor cells [24]. Our clinical findings could be explained by the latter results (Table 4). However, further studies have to validate our findings.

**Table 4:** Regression analysis for clinical risk factors

Variables	Positive surgical margin (R1)	Lymph node metasasis (N1)
	OR/*p* value	OR/*p* value
Age (years)	0.987/0.334	1.623/0.105
PSA (ng/ml)	0.848/0.397	1.140/0.255
Gleason score (6–10)	1.037/0.300	*3.454/0.001*
CAPRA score (1–5)	*2.916/0.004*	*3.200/0.001*

*PSA* prostate specific antigen, *CAPRA* Cancer of the Prostate Risk Assessment; *OR* odds ratio; italic = statistically significant OR

Also, beta-adrenergic signaling has been associated with advanced prostate cancer [25] and beta-blockers have been associated reduced mortality in various tumor types [25]. The prostate, especially the peripheral zone where most of the tumors originate, shows high adrenergic innervation [25]. Especially, beta2 receptor subtypes were detected in the peripheral zone. This could explain a possible protective effect by unselective beta-blockage such as carvedilol; however, the effect in our analysis was statistically not significant. The luminal epithelial cells which are suspected as the originating cells for prostate cancer show high expression for beta-adrenergic receptors in the malignant and benign state and thus connect the nervous system to the tumor [26]. Magnon et al. [27] showed that beta-adrenergic and cholinergic nerves play an important role in prostate cancer genesis and progression, by actively infiltrating the tumor. Perineural nerve sheath infiltration by the tumor also serves as an independent negative predictor for disease free survival [28]. Furthermore, beta-blockers were associated, we improved survival in prostate cancer patients [29]. Even though beta-blockers seem to have protective effects on long term survival, the underlying disease with an activated adrenergic system could well explain the increased risk for R1 disease in prostate cancer patients in our cohort. Further studies will have validate our results. Only scarce data is available upon alpha-adrenergic signaling and cancer. Some studies show inhibitory effects on cancer cells by alpha-adrenergic blockage [30–32].

Limitation of this study is the fact that comorbidities were defined only by self-medication. Additional comorbidities not reflected by the medication were not analyzed. Furthermore, dosage and duration of the medications were not considered.

We were able to show that together with the established stratification markers such as Gleason score or the CAPRA score, medication profiles can further aid in identifying men at high risk for advanced and aggressive prostate cancer (Table 5).

**Table 5:** Medication characteristics per patient

Number of mediaction systems/patient	
mean/±SD/median/IQR	2.544/2.059/2.0/1–4
Number of medications/patient	
mean/±SD/median/IQR	2.7/2.322/2.0/1–4
Number of medications/patient	
% (*n*/total)	
10–15	1.1 (5/454)
4–9	29.7 (135/454)
1–3	54.2 (246/454)
0	14.9 (68/454)

## Conclusions

Diabetes and beta-blockage are major risk factors for advanced prostate cancer, which should be incorporated into the pretherapeutic staging strategy and into the planning of definite therapy to allow for optimal results for our patients. The usage of electronic patient charts represent a powerful tool to analyze risk structures within patient cohorts. They could also be used to incorporate medication-based risk factors, which will automatically alert the physicians for high-risk patients.

## Additional file

Additional file 1: Medication characteristics (*n*=157). (DOCX 20 kb)
