# Supplementary material for: Diabetes and beta-adrenergic blockage are risk factors for metastatic prostate cancer
Source: World J Surg Oncol. 2017 Feb 21;15:50. doi: 10.1186/s12957-017-1117-4 (PMC5320736; doi:10.1186/s12957-017-1117-4)
Supplement: Additional file 1: — Medication characteristics (n=157). (DOCX 20 kb) [file 12957_2017_1117_MOESM1_ESM.docx]

Additional file 1

| Supplement Table 1 |  |  |
| --- | --- | --- |
| **CAPRA Score** |  |  |
| **Variable** |  | **Score** |
| PSA at diagnosis (ng/ml) | | |
| < 6 | | 0 |
| 6-10 | | 1 |
| 10-20 | | 2 |
| 20-30 | | 3 |
| >30 | | 4 |
| Gleason Pattern of the biopsy primary/secondary | | |
| 1-3/1-3 | | 0 |
| 1-3/4-5 | | 1 |
| 4-5/1-5 | | 3 |
| Age (years) | | |
| > 50 | | 0 |
| < 50 | | 1 |
| clinical tumor stage | | |
| T1a - T2c | | 0 |
| T3a | | 1 |
| positive biopsy cores (%) | | |
| < 33 | | 0 |
| >33 | | 1 |

Supplement Table 2

|  |  |
| --- | --- |
| **medication characteristics (n=157)** | |
| **Name** | **ATC Code** |
| alimentary tract | |
| Dexamethason | A01AC02 |
| Hydrocortison | A01AC03 |
| Famotidin | A02BA03 |
| Omeprazol | A02BC01 |
| Pantoprazol | A02BC02 |
| Rabeprazol | A02BC04 |
| Esomeprazol | A02BC05 |
| Sucralfat | A02BX02 |
| Atropin | A03BA01 |
| Scopolamin | A03BB01 |
| Prednisolon | A07EA01 |
| Budesonid | A07EA06 |
| Beclometason | A07EA07 |
| Insulin | A10AB01 |
| Metformin | A10BA02 |
| Glibenclamid | A10BB01 |
| Gliclazid | A10BB09 |
| Glimepirid | A10BB12 |
| Metformin/Sigaliptin | A10BH01 |
| Sitagliptin | A10BH01 |
| Vildagliptin | A10BH02 |
| blood system |  |
| phenprocoumon | B01AA04 |
| Clopidogrel | B01AC04 |
| Acetylsalicacid | B01AC06 |
| dipyridamol | B01AC07 |
| Prasugrel | B01AC22 |
| cardiovascular system |  |
| Digitoxin | C01AA04 |
| Digoxin | C01AA05 |
| Flecanaid | C01BC04 |
| Amiodaron | C01BD01 |
| Midodrin | C01CA17 |
| Isorbid | C01DA08 |
| Ubidecarenon | C01EB09 |
| Ivabradin | C01EB17 |
| Clonidin | C02AC01 |
| Moxonidin | C02AC05 |
| Doxazosin | C02CA04 |
| Urapidil | C02CA06 |
| Dihydralazin | C02DB01 |
| hydrochlorothiazid | C03AA03 |
| Xipamid | C03BA10 |
| Furosemid | C03CA01 |
| Torasemid | C03CA04 |
| Spironolacton | C03DA01 |
| Eplerenon | C03DA04 |
| Amilorid | C03DB01 |
| Triamteren | C03DB02 |
| Pindolol | C07AA03 |
| Sotalol | C07AA07 |
| Metoprolol | C07AB02 |
| Atenolol | C07AB03 |
| Betaxolol | C07AB05 |
| Bisoprolol | C07AB07 |
| Nebivolol | C07AB12 |
| Carvedilol | C07AG02 |
| Dihydropyridin | C08CA |
| Amlodipin | C08CA01 |
| Felodipin | C08CA02 |
| Nifedipin | C08CA05 |
| Nitrendipin | C08CA08 |
| Lercanidipin | C08CA13 |
| Verapamil | C08DA01 |
| Captopril | C09AA01 |
| Enalapril | C09AA02 |
| Lisinopril | C09AA03 |
| Perindopril | C09AA04 |
| Ramipril | C09AA05 |
| Spirapril | C09AA11 |
| Losartan | C09CA01 |
| Valsartan | C09CA03 |
| Irbesartan | C09CA04 |
| Candesartan | C09CA06 |
| Telmisartan | C09CA07 |
| Olmesartan | C09CA08 |
| Hydrochlorthiazid/Telmisartan | C09DA07 |
| aliskiren | C09XA02 |
| Simvastatin | C10AA01 |
| Lovastatin | C10AA02 |
| Pravastatin | C10AA03 |
| Fluvastatin | C10AA04 |
| Atorvastatin | C10AA05 |
| Rosuvastatin | C10AA07 |
| Fenofibrat | C10AB05 |
| Colestyramin | C10AC01 |
| Ezetemib | C10AX09 |
| genitourinary system |  |
| Propiverin | G04BD06 |
| Tolterodin | G04BD07 |
| Solifenacin | G04BD08 |
| Trospium | G04BD09 |
| Fesoterodin | G04BD11 |
| Alfuzosin | G04CA01 |
| Tamsulosin | G04CA02 |
| Finasterid | G04CB01 |
| hormonal system |  |
| Levothyroxin | H03AA01 |
| Liothyronin | H03AA02 |
| Perchlorat | H03BC01 |
| immune system |  |
| Mycophenolat | L04AA06 |
| Tacrolimus | L04AD02 |
| nervous system |  |
| Primidon | N03AA03 |
| Lamotrigin | N03AX09 |
| Gabapentin | N03AX12 |
| Levetiracetam | N03AX14 |
| Pregabalin | N03AX16 |
| Levodopa | N04BA01 |
| Benserazid | N04BA02 |
| Carbidopa | N04BA03 |
| Ropinirol | N04BC04 |
| Pramipexol | N04BC05 |
| Thioridazin | N05AC02 |
| Chlorprothixen | N05AF03 |
| Quetiapin | N05AH04 |
| Lithium | N05AN01 |
| Risperidon | N05AX08 |
| Bromazepam | N05BA08 |
| Midazolam | N05CD08 |
| Zopiclon | N05CF01 |
| Zolpidem | N05CF02 |
| Trimipramin | N06AA06 |
| Amytriptylin | N06AA09 |
| Doxepin | N06AA12 |
| Fluoxetin | N06AB03 |
| Citalopram | N06AB04 |
| Paroxetin | N06AB05 |
| Sertralin | N06AB06 |
| Escitalopram | N06AB10 |
| Tranylcypromin | N06AF04 |
| Mirtazapin | N06AX11 |
| Duloxetin | N06AX21 |
| Piracetam | N06BX03 |
| Rivastigmin | N06DA03 |
| Betahistin | N07CA01 |
| respiratory system |  |
| Phenylephrin | R01AA04 |
| Budesonid | R01AD05 |
| Fluticason | R01AD08 |
| Salbutamol | R03AC02 |
| Terbutalin | R03AC03 |
| Fenoterol | R03AC04 |
| Salmeterol | R03AC12 |
| Formoterol | R03AC13 |
| Indacaterol | R03AC18 |
| Ipratropium | R03BB01 |
| Tiotropium | R03BB04 |
| Aclidinium bromid | R03BB05 |
| Theophyllin | R03DA04 |
| Montelukast | R03DC03 |
| sensory system |  |
| Acetazolamid | S01EC01 |
| Dorzolamid | S01EC03 |
| Metipranolol | S01ED04 |
| Latanoprost | S01EE01 |
| Tropicamid | S01FA06 |
| Hypromellose | S01KA02 |
